# Comprehensive understanding of the adverse effects associated with temozolomide: a disproportionate analysis based on the FAERS database

**DOI:** 10.3389/fphar.2024.1437436

**Published:** 2024-08-23

**Authors:** Yusen Zhou, Peng Jia, Yuting Fang, Wei Zhu, Yong Gong, Tianyu Fan, Jiangliu Yin

**Affiliations:** ^1^ Department of Neurosurgery, The Affiliated Changsha Central Hospital, University of South China, Changsha, Hunan, China; ^2^ Department of Surgery, 94750th Hospital of Chinese People’s Liberation Army, Longyan, Fujian, China

**Keywords:** temozolomide, FAERS, pharmacovigilance, real-world data analysis, adverse effects, glioma

## Abstract

**Background:**

Temozolomide, which is the standard drug for glioma treatment, has several Adverse events (AEs) in the treatment of gliomas and other tumors that are not yet fully understood. This is due to the pharmacological nature of the alkylating agent. A significant proportion of these effects have not been systematically documented or reported.

**Methods:**

We selected data from the United States FDA Adverse Event Reporting System (FAERS) database from the first quarter of 2004 to the fourth quarter of 2023. Four algorithms were used for disproportionate analysis, with the objective of assessing the association between temozolomide and related adverse events.

**Results:**

In this study, 20,079,906 case reports were collected from the FAERS database, of which 15,152 adverse events related to temozolomide were reported. A total of 352 preferred terms (PTs) and 24 system organ classes (SOCs) that were significantly disproportionally related to the four algorithms were included. The SOCs included blood and lymphatic system disorders (χ^2^ = 18,220.09, n = 4,325); skin and subcutaneous tissue disorders (χ^2^ = 408.06, n = 1,347); investigations (χ^2^ = 639.44, n = 3,925); musculoskeletal and connective tissue disorders (χ^2^ = 1,317.29, n = 588); and psychiatric disorders (χ^2^ = 1,098.47, n = 877). PT levels were screened for adverse drug reaction signals consistent with drug inserts, such as anemia, thrombocytopenia, liver function abnormalities, nausea and vomiting, as well as rarely reported adverse drug reactions, such as aplastic anemia, myelodysplastic syndromes, electrolyte disorders, cerebral edema, and high-frequency mutations.

**Conclusion:**

The results of our investigation demonstrated both adverse effects that had been reported and a multitude of unreported adverse effects that were serious in nature and lacked a clear cause. These novel findings suggest that more attention should be given to the clinical conditions of patients after treatment to provide a more comprehensive perspective and understanding for further clarifying the safety of temozolomide.

## 1 Introduction

Temozolomide (TMZ) is an alkylate from the imidazotetrazine family and is the standard treatment for glioblastoma multiforme (Lee, 2016). TMZ was originally developed to treat primary brain tumors and was approved by the FDA and EMEA in 1999 for the treatment of glioblastoma multiforme and anaplastic astrocytoma. The benefit of TMZ in the treatment of high-grade glioma is clear. According to the European Organization for Research and Treatment of Cancer (EORTC) and National Cancer Institute of Canada Clinical Trials Group (NCIC) study, postoperative radiotherapy combined with TMZ is associated with a better prognosis than adjuvant radiotherapy alone, and patients with 1p/19q noncodeletion and O6-methylguanine-DNA methyltransferase (MGMT) methylation status are most likely to benefit from the addition of TMZ (Stupp et al., 2009; van den Bent et al., 2017). In addition, TMZ has shown considerable value in low-grade gliomas because of its significant radiostability and ability to delay the demand for radiotherapy (Wahl et al., 2017). With extensive research on the mechanism and effect of TMZ, the role of TMZ in the treatment of other tumors has also been confirmed. TMZ is a derivative of the imidazotetrazine derivative dacarbazine, which is the most commonly used chemotherapy agent in metastatic melanoma. TMZ has been proven to have an equal efficacy to dacarbazine in a randomized phase III trial conducted in patients with melanoma (Middleton et al., 2000). Furthermore, TMZ was established as being a first-line chemotherapy for pituitary tumors and pituitary carcinomas in the 2018 ESE guidelines (McCormack, 2022). The role of TMZ in neuroendocrine tumors, non-small cell lung cancer, pancreatic tumors, Ewing’s sarcoma and other diseases has also been mentioned in some studies (Tatar et al., 2013). In the treatment of ovarian cancer, the combination of temozolomide and a PARP inhibitor exploits the specific DNA damage repair status of ARID1A-inactivated ovarian cancers to suppress tumor growth (Yu et al., 2023).

Although TMZ exhibits significant therapeutic value, its inherent toxicity as an alkylating agent should not be underestimated. The metabolic response of TMZ is pH dependent, and TMZ is hydrolyzed *in vivo* to 5-(3-methyltriazen-1-yl) imidazole-4-carboxamide (MTIC), which is subsequently broken down to 5-aminoimidazole-4-carboxamide (AIC) and methyl diazide cation, which methylates DNA and consequently causes apoptosis to achieve tumor therapy (Friedman et al., 2000). However, the short half-life of MTIC and its weak ability to cross the blood‒brain barrier often makes it necessary for patients to take high doses of TMZ to ensure efficacy, which has potential for eliciting severe nonspecific toxicity (Fang et al., 2015). Adverse events (AEs) from TMZ have the potential to negatively impact patient quality of life. According to one report, among the hematologic AEs, thrombocytopenia, anemia, and increased AST/ALT were commonly reported due to this treatment. Among the nonhematologic AEs, nausea, vomiting, and anorexia were the three most common AEs (Bae et al., 2014). A systematic review reported of several uncommon AEs of TMZ, such as cholestatic hepatitis, pneumonia, and other opportunistic infections (Dixit et al., 2012). These AEs have attracted the attention of researchers. Therefore, a comprehensive collection and analysis of AEs to TMZ is necessary to evaluate the balance of treatment benefits and damage. As researchers have focused on the limitations of TMZ, such as poor hydrolysis and solubility, tumor heterogeneity, and therapeutic resistance, alterations in drug encapsulation has become a new treatment option. The use of nanomaterials to encapsulate TMZ to improve its stability, especially using polymeric and lipid-based nanosystems, can improve TMZ resistance and increase its solubility, blood circulation and biodistribution, in addition to enhancing blood‒brain barrier penetration (Tian et al., 2011; Iturrioz-Rodríguez et al., 2023). Therefore, the comprehensive collection and analysis of the AEs of TMZ are beneficial for providing current researchers with a comprehensive reference for promoting improvements in TMZ development and improving its efficacy.

Current research on the AEs of TMZ is primarily based on clinical trials, which lack a global, real-world-based understanding of the adverse effects of the drug due to the stringent access conditions of clinical trials. The FDA Adverse Event Reporting System (FAERS) is a database that contains information on AEs associated with the use of drugs. It can be used to identify potential drug safety signals and to assess the safety of drugs. Numerous studies have demonstrated the feasibility and usefulness of FAERS for monitoring AEs associated with the use of drugs in real-world settings. For example, in Du’s investigation, the application of data mining methodologies to the FAERS database demonstrated unreported AEs, including acute pancreatitis, associated with metformin (Du et al., 2024). Therefore, this study aimed to statistically and analytically evaluate the real-world AEs of TMZ after its launch through data mining methods on the FAERS database, thus aiming to increase patient safety during the course of TMZ therapy by promoting more comprehensive and effective AE monitoring and management frameworks and providing a more comprehensive basis for subsequent clinical treatments and drug studies.

## 2 Methods

### 2.1 Data sources and preprocessing

**FIGURE 1:**
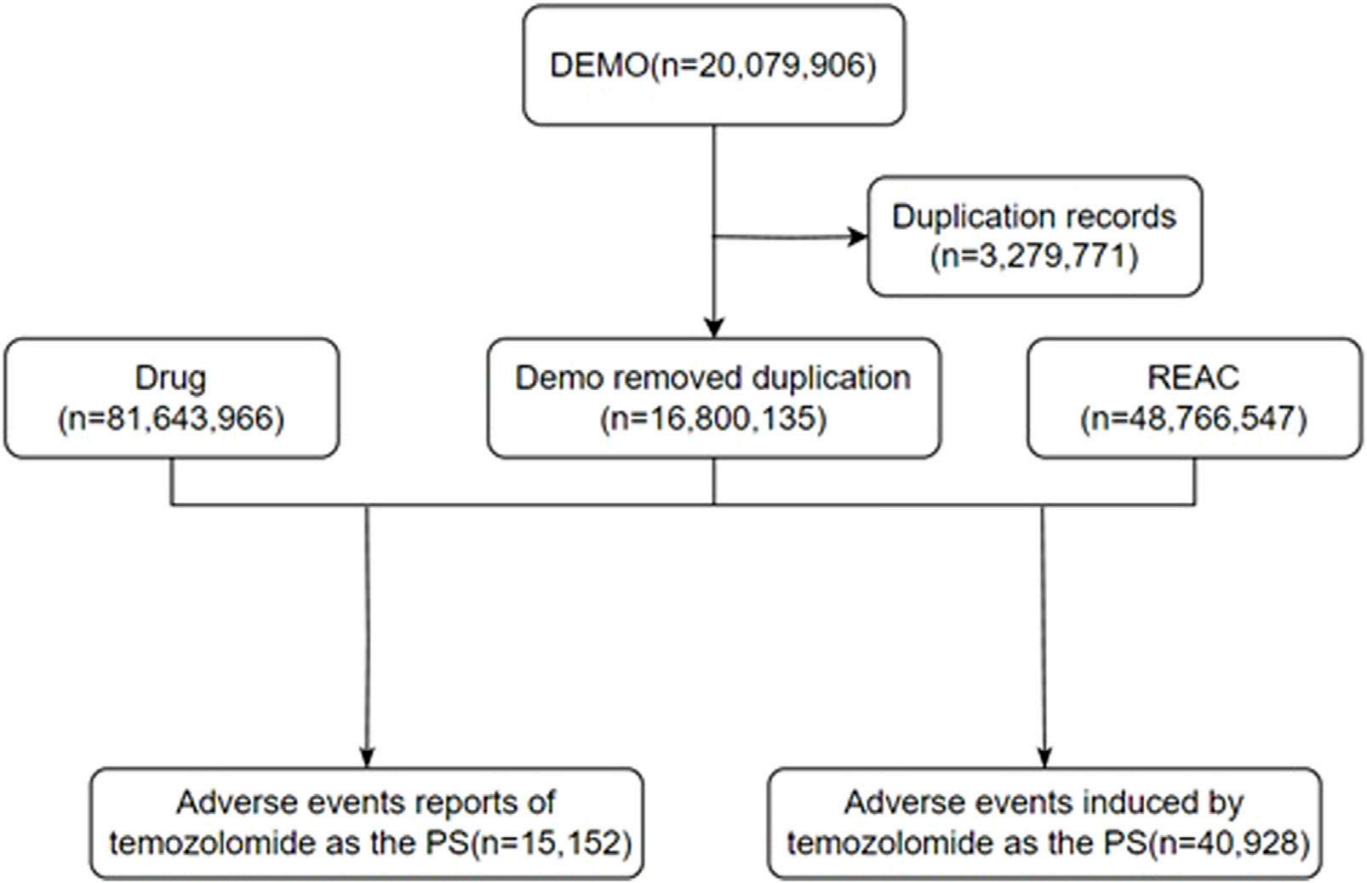
The flow diagram of selecting TMZ-related AEs from the FAERS database. DEMO: demographic; REAC, reaction; PS, primary suspect.

In this study, a retrospective pharmacovigilance analysis was performed on the data on AEs related to TMZ in the FAERS database from 2004 to 2023. Due to the uniqueness of the data structure of the FAERS database, all of the data are presented in a quarterly summary format, which includes AEs, medication error reports, and product quality complaints. The extracted data from the official website (https://fis.fda.gov/extensions/FPD-QDE-FAERS/FPD-QDE-FAERS.html) were organized and analyzed by using Excel (office 2020) and R STUDIO software (2023.03. 0 + 386.pro1). The data were thoroughly cleaned and standardized; moreover, duplicates were removed. Adverse drug reactions (ADRs) were determined by using statistical methods. This study collected ADRs associated with TMZ over the last 20 years and summarized and categorized them according to the different preferred term levels (PTs) and system organ class levels (SOCs) in the Medical Dictionary for Regulatory Activities (MedDRA). The general flowchart of this study is shown in Figure 1.

TMZ was used in this study as our primary suspected (PS) drug for identifying ADRs in the FAERS database. Baseline factors, such as sex, age, reporter, reporting country or region, ADR outcome, duration of drug administration, and time of disease onset, were summarized in the search results for a more complete understanding of the data; additionally, serious ADR outcomes included hospitalization, death, incapacitation, and other life-threatening serious ADR outcomes. For each year of ADR, we used line graphs for presentation to observe the time trend of ADRs. For indications of TMZ, pie graph was used to present the application of TMZ.

### 2.2 Statistical analysis

To further explore the association between AEs and TMZ, we used the frequency method of disproportionality analysis (DPA) based on 2 × 2 columnar tables, which includes reporting of dominance ratios (RORs) and proportional reporting ratios (PRRs), in order to evaluate the frequency of observations in the drug-using population in relation to the non-using population. Higher values of the RORs and the PRRs indicated stronger signals of the AEs, which suggests a stronger association between the target drug and the AEs. In addition, we used the Bayesian confidence propagation neural network (BCPNN) and empirical Bayesian geometric mean (EBGM) methods for complementary analysis to reduce the generation of false-positive AE signals. The criteria for positive safety signal detection were an ROR ≥3, a PRR ≥2, and an EBGM05 >2 (EBGM05: lower limit of the 95% confidence interval).

## 3 Results

### 3.1 Basic information about adverse events associated with temozolomide

**FIGURE 2:**
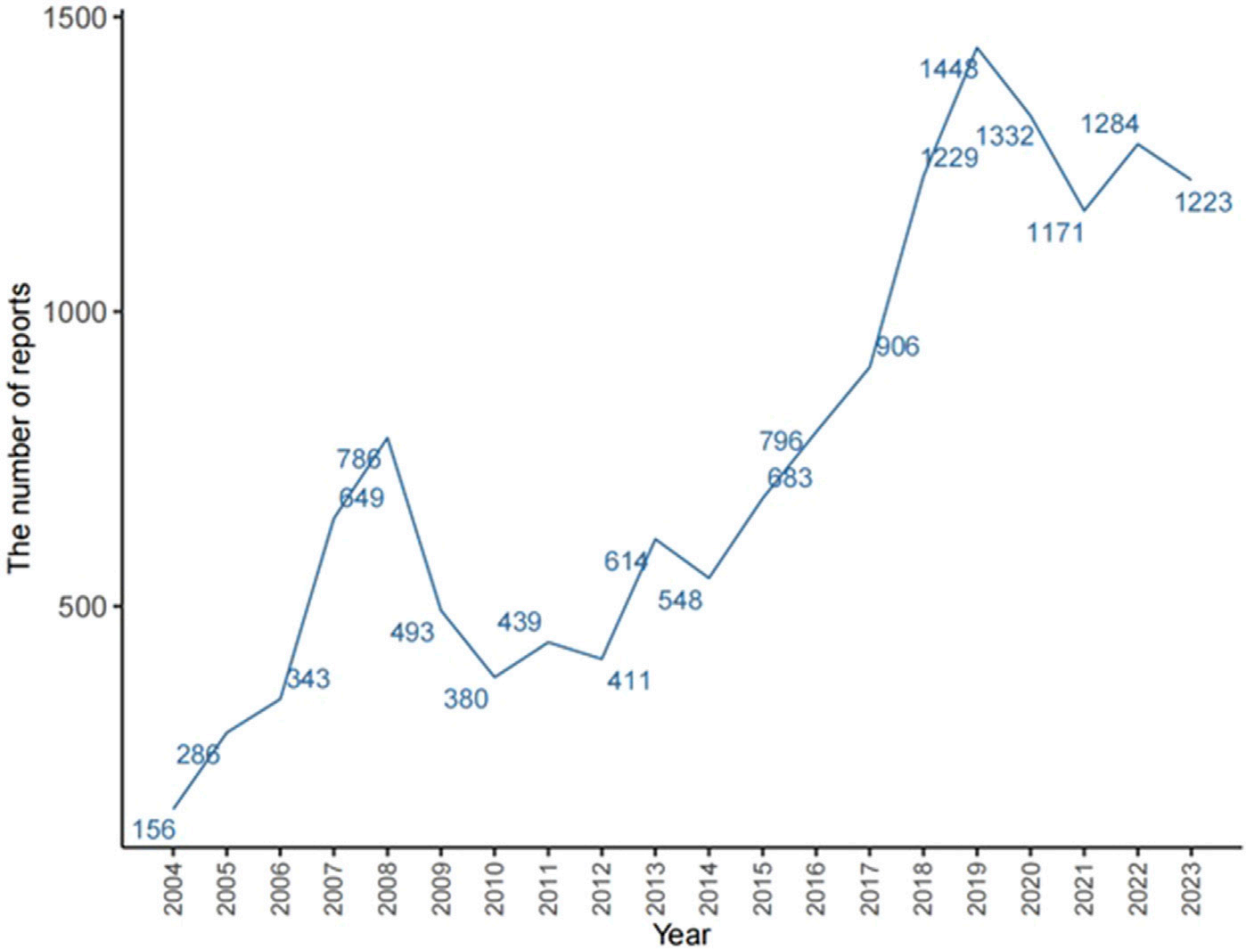
Number of AEs reported annually since TMZ launch.

The total number of ADRs extracted from the FAERS from 2004 to 2023 was 20,079,906; after eliminating 3,279,771 duplicate reports, 15,152 ADRs associated with TMZ were screened, of which 40,928 ADRs associated with TMZ combined with PS were identified. As shown in Figure 2, the number of ADRs showed an overall increasing trend over time. Table 1 shows the relevant baseline characteristics in detail, with a greater proportion of males (53.65%) than females (46.35%) reporting of AEs. In terms of age, the incidence of AEs was greater in the 60 years and above age group (34.55%) than in the other age groups; notably, the incidence of AEs in the 40–60 years age group was also 28.3%, which suggests that there is an association between the incidence of AEs related to TMZ and sex, age, and possibly glioma. Regionally, the United States (50.46%) had the most reports, followed by Japan (3.73%) and Canada (3.4%). Despite the predominance of the United States as the reporting country, all of the top-ranking countries were developed countries. The study demonstrated that the incidence of tumors increased with the Human Development Index (HDI), with populations in countries with high HDI levels having significantly greater incidence rates than those in countries with low HDI levels (Sung et al., 2021). The reporters were mainly physicians (30.45%) and pharmacists (27.64%); moreover, according to the database analysis, the most serious AEs of TMZ were hospitalization, death, life-threatening disease and disability, and other unknown serious outcomes. In addition to unknown serious ADRs (34.24%), hospitalization (35.78%) was the most common serious adverse outcome, with 4,841 cases being reported, followed by death and life-threatening events, with 3,082 (22.78%) and 706 (5.22%) cases, respectively.

**TABLE 1:** Clinical characteristics of TMZ reported as a primary suspected drug in the FAERS database (2004Q1–2023Q4).

Variable	Temozolomide	
	Counts, n = 13,602	Percentage
Sex		
Female	6,305	46.35
Male	7,297	53.65
Age		
<20	797	5.86
20∼40	1,400	10.29
40∼60	3,850	28.3
≥60	4,700	34.55
Unknow	2,855	20.99
Reporter		
Physician	4,142	30.45
Pharmacist	3,759	27.64
Unknown	2,098	15.42
Other health-professional	1,899	13.96
Consumer	1,688	12.41
Registered Nurse	16	0.12
Reported countries		
United States	6,864	50.46
Other	4,398	32.33
Japan	508	3.73
Canada	462	3.4
France	423	3.11
Germany	270	1.99
United Kingdom	241	1.77
Italy	212	1.56
China	114	0.84
Spain	110	0.81
Route		
Oral	8,591	63.15
Other	4,820	35.43
Intravenous drip	95	0.7
Intravenous	87	0.64
Transplacental	12	0.09
Outcomes		
Hospitalization	4,841	35.78
Other serious	4,633	34.24
Death	3,082	22.78
Life threatening	706	5.22
Disability	218	1.61
Required intervention to Prevent Permanent Impairment/Damage	37	0.27
Congenital anomaly	12	0.09
Weight	73.60 (60.00, 88.00)	
Time to onset	45.00 (20.00, 140.00)	

### 3.2 Signal detection based on system organ class levels

The signal strength and reporting frequency of TMZ at the SOC level are presented in Table 2. AEs related to TMZ occurred in all 24 target SOCs, as shown in Table 2. Among the four analyzed algorithms, at least one significant SOC was identified as meeting the criteria, including blood and lymphatic system disorders (χ^2^ = 18,220.09, n = 4,325); skin and subcutaneous tissue disorders (χ^2^ = 408.06, n = 1,347); investigations (χ^2^ = 639.44, n = 3,925); musculoskeletal and connective tissue disorders (χ^2^ = 1,317.29, n = 588); and psychiatric disorders (χ^2^ = 1,098.47, n = 877). In terms of volume, the top ten SOCs included general disorders and administration site conditions; blood and lymphatic system disorders; investigations; nervous system disorders; gastrointestinal disorders; injury, poisoning and procedural complications; infections and infestations; benign, malignant and unspecified neoplasms; respiratory, thoracic and mediastinal disorders; and skin and subcutaneous tissue disorders.

**TABLE 2:** The signal strength of the AEs of TMZ at the SOC level in the FAERS database.

SOC	Case reports	ROR (95% CI)	PRR (95% CI)	Chi square	IC(IC025)	EBGM(EBGM05)
Blood and lymphatic system disorders	4,325	6.58 (6.38, 6.79)	5.99 (5.87, 6.11)	18,220.09	2.58 (2.53)	5.97 (5.81)
Neoplasms benign, malignant and unspecified	2,637	2.37 (2.28, 2.46)	2.28 (2.19, 2.37)	1,944.58	1.19 (1.13)	2.28 (2.2)
Hepatobiliary disorders	784	2.03 (1.89, 2.18)	2.01 (1.86, 2.17)	402.22	1.01 (0.91)	2.01 (1.89)
Congenital, familial and genetic disorders	266	2 (1.77, 2.26)	1.99 (1.77, 2.24)	131.74	0.99 (0.82)	1.99 (1.8)
Investigations	3,925	1.52 (1.48, 1.58)	1.47 (1.41, 1.53)	639.44	0.56 (0.51)	1.47 (1.43)
Infections and infestations	2,916	1.33 (1.28, 1.38)	1.31 (1.26, 1.36)	222.14	0.39 (0.33)	1.31 (1.27)
Metabolism and nutrition disorders	1,207	1.32 (1.25, 1.4)	1.31 (1.24, 1.39)	90.99	0.39 (0.31)	1.31 (1.25)
Endocrine disorders	128	1.19 (1, 1.42)	1.19 (1, 1.42)	4	0.25 (0)	1.19 (1.03)
Nervous system disorders	3,833	1.05 (1.02, 1.09)	1.05 (1.01, 1.09)	9.36	0.07 (0.02)	1.05 (1.02)
Gastrointestinal disorders	3,569	0.97 (0.94, 1.01)	0.98 (0.94, 1.02)	2.42	−0.04 (−0.09)	0.98 (0.95)
General disorders and administration site conditions	7,226	0.97 (0.94, 0.99)	0.97 (0.95, 0.99)	6.84	−0.04 (−0.08)	0.97 (0.95)
Vascular disorders	895	0.96 (0.9, 1.02)	0.96 (0.91, 1.02)	1.65	−0.06 (−0.16)	0.96 (0.91)
Respiratory, thoracic and mediastinal disorders	1,709	0.83 (0.79, 0.87)	0.84 (0.81, 0.87)	56.79	−0.26 (−0.33)	0.84 (0.8)
Injury, poisoning and procedural complications	3,047	0.78 (0.75, 0.8)	0.79 (0.76, 0.82)	182.86	−0.34 (−0.39)	0.79 (0.77)
Skin and subcutaneous tissue disorders	1,347	0.58 (0.54, 0.61)	0.59 (0.56, 0.63)	408.06	−0.76 (−0.84)	0.59 (0.56)
Ear and labyrinth disorders	104	0.56 (0.46, 0.68)	0.56 (0.46, 0.68)	35.55	−0.83 (−1.11)	0.56 (0.48)
Renal and urinary disorders	416	0.52 (0.47, 0.57)	0.52 (0.47, 0.57)	184.61	−0.93 (-1.07)	0.52 (0.48)
Immune system disorders	230	0.49 (0.43, 0.55)	0.49 (0.43, 0.56)	124.95	−1.03 (−1.22)	0.49 (0.44)
Cardiac disorders	543	0.46 (0.42, 0.5)	0.47 (0.43, 0.51)	337.04	−1.09 (−1.22)	0.47 (0.44)
Psychiatric disorders	877	0.34 (0.32, 0.36)	0.35 (0.33, 0.37)	1,098.47	−1.5 (−1.59)	0.35 (0.34)
Eye disorders	262	0.3 (0.27, 0.34)	0.31 (0.28, 0.35)	417.7	−1.7 (−1.88)	0.31 (0.28)
Musculoskeletal and connective tissue disorders	588	0.25 (0.23, 0.27)	0.26 (0.24, 0.28)	1,317.29	−1.95 (−2.06)	0.26 (0.24)
Pregnancy, puerperium and perinatal conditions	42	0.22 (0.17, 0.3)	0.22 (0.16, 0.3)	113.22	−2.16 (−2.59)	0.22 (0.17)
Reproductive system and breast disorders	52	0.14 (0.11, 0.19)	0.15 (0.11, 0.2)	264.26	−2.78 (−3.17)	0.15 (0.12)

### 3.3 Signal detection based on preferred term levels

After establishing the four methods, we retrieved 352 cases of AE signaling associated with TMZ. Table 3 shows that 50 patients had the highest number of cases (Table 3). Of these, the PT with the highest number of reported cases was disease progression (n = 1,265), followed by thrombocytopenia (n = 1,078). Among the hematologic and lymphatic disorders, the PT results for anemia, febrile neutropenia, leukopenia, and thrombocytopenia were consistent with the specification, and we also detected signals for pancytopenia (n = 501), bone marrow failure (n = 279), myelosuppression (n = 60), and lymphocytopenia (n = 242) in PTs. In tumors, the signal intensity of tumor progression (n = 808), tumor recurrence (n = 74), and myelodysplastic syndrome (n = 58) was also significant, which was similar to what was documented in the manual. Among neurological disorders, epileptic seizures (n = 412), cerebral edema (n = 198), unilateral hemiplegia (n = 119), aphasia (n = 105), cerebral hemorrhage (n = 92), hydrocephalus (n = 71), and epilepsy (n = 63) were identified; moreover, except for hemiplegia, the other PT signals were not well documented in the manual. On examination, we detected decreased platelet count (n = 605), decreased white blood cell count (n = 335), decreased lymphocyte count (n = 194), decreased neutrophil count (n = 173), increased Alt (n = 172), increased Ast (n = 121), and increased GGT (n = 69) AE signals. The first four AE signaling results were consistent with those of the blood system; moreover, it has been observed that TMZ-associated AEs potentially affected liver function, such as by causing elevated Alt, Ast, and GGT. In metabolic and nutritional disorders, in addition to hyperglycemia mentioned in the specification, we demonstrated dehydration, hyponatremia, and hypokalemia, which were not documented in the specification, although AEs mentioning loss of appetite in the specification were not retrieved. Therefore, we hypothesized that loss of appetite may be associated with severe dehydration and that hyponatremia is often observed in hypotonic dehydration, which can contribute to the development of other AEs, such as loss of appetite. In infectious diseases, the signal of sepsis (n = 256) was particularly significant, in addition to the AE signal of infectious shock (n = 73), whereas severe sepsis is highly likely to lead to infectious shock. For respiratory diseases, we retrieved AE signals for pulmonary embolism, which was not mentioned in the specification. We also identified PTs such as deep vein thrombosis (n = 209), petechiae (n = 68), and high-frequency mutations (n = 88) that were not registered in the manual and were significantly different from those described in the manual.

**TABLE 3:** The top 50 corresponding PTs for case reports in the FAERS database were selected based on 352 PTs that met the four algorithmic criteria.

SOC	PT	Case reports	ROR (95% CI)	PRR (95% CI)	Chisq	IC (IC025)	EBGM (EBGM05)
Blood and lymphatic system disorders	Thrombocytopenia	1,078	14.26 (13.42, 15.15)	13.91 (13.12, 14.75)	12,789.13	3.78 (3.69)	13.76 (13.08)
	Neutropenia	533	5.79 (5.32, 6.31)	5.73 (5.3, 6.2)	2076.07	2.51 (2.39)	5.71 (5.31)
	Pancytopenia	501	13.18 (12.06, 14.4)	13.03 (12.05, 14.09)	5,509.91	3.69 (3.56)	12.9 (11.98)
	Febrile neutropenia	371	8.33 (7.52, 9.23)	8.26 (7.49, 9.11)	2,353.8	3.04 (2.89)	8.21 (7.54)
	Anemia	338	2.45 (2.2, 2.73)	2.44 (2.21, 2.69)	288.02	1.29 (1.13)	2.44 (2.23)
	Bone marrow failure	279	17.86 (15.87, 20.11)	17.75 (15.78, 19.97)	4,346.36	4.13 (3.96)	17.5 (15.85)
	Lymphopenia	242	25.23 (22.21, 28.67)	25.09 (22.31, 28.22)	5,482.66	4.62 (4.44)	24.59 (22.1)
	Leukopenia	228	6.53 (5.73, 7.44)	6.5 (5.67, 7.46)	1,056.9	2.69 (2.51)	6.47 (5.8)
	Aplastic anemia	144	40.51 (34.3, 47.84)	40.37 (34.51, 47.22)	5,347.47	5.29 (5.05)	39.08 (34)
	Hepatotoxicity	110	19.17 (15.88, 23.15)	19.12 (15.72, 23.26)	1859.73	4.24 (3.96)	18.84 (16.09)
	Agranulocytosis	60	4.9 (3.8, 6.31)	4.89 (3.79, 6.31)	185.19	2.29 (1.92)	4.88 (3.94)
	Myelosuppression	60	4.96 (3.85, 6.39)	4.95 (3.84, 6.39)	188.64	2.3 (1.94)	4.94 (3.99)
Neoplasms benign, malignant and unspecified	Malignant neoplasm progression	518	7.65 (7.02, 8.35)	7.57 (7, 8.19)	2,940.11	2.91 (2.79)	7.53 (7)
	Neoplasm progression	290	11.33 (10.09, 12.72)	11.25 (10, 12.65)	2,685.56	3.48 (3.31)	11.16 (10.12)
	Neoplasm recurrence	74	31.7 (25.16, 39.94)	31.64 (25.01, 40.03)	2,139.3	4.95 (4.62)	30.85 (25.43)
	Myelodysplastic Syndrome	58	5.54 (4.28, 7.17)	5.53 (4.29, 7.13)	214.42	2.46 (2.09)	5.51 (4.44)
Nervous system disorders	Seizure	412	5.51 (5, 6.07)	5.47 (4.96, 6.03)	1,499.26	2.45 (2.3)	5.45 (5.02)
	Brain edema	198	22.36 (19.42, 25.74)	22.26 (19.41, 25.53)	3,946.87	4.45 (4.25)	21.87 (19.43)
	Hemiparesis	119	9.41 (7.85, 11.27)	9.38 (7.86, 11.19)	884.48	3.22 (2.96)	9.32 (8.01)
	Aphasia	105	4.77 (3.94, 5.78)	4.76 (3.91, 5.79)	311.14	2.25 (1.97)	4.75 (4.04)
	Cerebral hemorrhage	92	3.56 (2.9, 4.37)	3.55 (2.92, 4.32)	168.26	1.83 (1.53)	3.54 (2.99)
	Hydrocephalus	71	18.4 (14.55, 23.26)	18.37 (14.52, 23.24)	1,148.35	4.18 (3.84)	18.1 (14.88)
	Epilepsy	63	3.03 (2.36, 3.88)	3.03 (2.35, 3.91)	85.26	1.59 (1.24)	3.02 (2.46)
Investigations	Platelet count decreased	606	8.12 (7.49, 8.8)	8.01 (7.41, 8.66)	3,700.03	2.99 (2.88)	7.96 (7.44)
	White blood cell count decreased	335	4.38 (3.93, 4.88)	4.35 (3.94, 4.8)	863.76	2.12 (1.96)	4.34 (3.97)
	Lymphocyte count decreased	194	15.4 (13.36, 17.75)	15.34 (13.37, 17.6)	2,567.39	3.92 (3.72)	15.15 (13.46)
	Neutrophil count decreased	173	6.32 (5.44, 7.35)	6.3 (5.39, 7.37)	768.11	2.65 (2.43)	6.27 (5.54)
	Alanine aminotransferase increased	172	3.83 (3.3, 4.45)	3.82 (3.27, 4.47)	356.94	1.93 (1.71)	3.81 (3.36)
	Aspartate aminotransferase increased	121	3.11 (2.6, 3.71)	3.1 (2.6, 3.7)	171.86	1.63 (1.37)	3.09 (2.66)
	Gamma-glutamyltransferase increased	69	4.12 (3.25, 5.22)	4.11 (3.25, 5.2)	162.03	2.04 (1.7)	4.1 (3.36)
General disorders and administration site conditions	Disease progression	1,265	16.28 (15.39, 17.23)	15.81 (14.91, 16.77)	17,355.99	3.97 (3.88)	15.62 (14.9)
	General physical health deterioration	204	2.75 (2.39, 3.15)	2.74 (2.39, 3.14)	225.09	1.45 (1.25)	2.73 (2.44)
	Disease recurrence	172	5.53 (4.76, 6.43)	5.51 (4.71, 6.45)	633.19	2.46 (2.24)	5.49 (4.84)
	Therapy partial responder	68	10.09 (7.95, 12.81)	10.08 (7.97, 12.75)	551.3	3.32 (2.98)	10 (8.19)
	Mucosal inflammation	62	3.41 (2.66, 4.38)	3.41 (2.64, 4.4)	105.35	1.77 (1.41)	3.4 (2.76)
Metabolism and nutrition disorders	Dehydration	226	2.35 (2.06, 2.67)	2.34 (2.04, 2.68)	173.31	1.22 (1.04)	2.34 (2.09)
	Hyponatremia	105	2.58 (2.13, 3.13)	2.58 (2.12, 3.14)	101.53	1.37 (1.09)	2.58 (2.2)
	Hyperglycemia	89	3.38 (2.75, 4.16)	3.38 (2.72, 4.19)	148.47	1.75 (1.45)	3.37 (2.83)
	Hypokalemia	84	2.6 (2.1, 3.23)	2.6 (2.1, 3.23)	82.66	1.38 (1.07)	2.6 (2.17)
Infections and infestations	Sepsis	256	3.22 (2.84, 3.64)	3.2 (2.84, 3.6)	387.47	1.68 (1.5)	3.2 (2.88)
	Aspergillus infection	74	17.94 (14.25, 22.57)	17.91 (14.16, 22.66)	1,163.81	4.14 (3.81)	17.66 (14.57)
	Septic shock	73	2.46 (1.96, 3.1)	2.46 (1.94, 3.11)	63.14	1.3 (0.97)	2.46 (2.03)
	Pneumonia aspiration	70	3.99 (3.15, 5.04)	3.98 (3.15, 5.04)	155.91	1.99 (1.65)	3.97 (3.26)
Skin and subcutaneous tissue disorders	Petechiae	68	9.11 (7.18, 11.57)	9.1 (7.19, 11.51)	486.66	3.18 (2.83)	9.04 (7.4)
Skin and subcutaneous tissue disorders	Drug reaction with eosinophilia and systemic symptoms	56	3.32 (2.56, 4.32)	3.32 (2.57, 4.28)	90.57	1.73 (1.35)	3.31 (2.66)
Vascular disorders	Deep vein thrombosis	209	4.23 (3.69, 4.85)	4.21 (3.67, 4.83)	511.08	2.07 (1.88)	4.2 (3.75)
Respiratory, thoracic and mediastinal disorders	Pulmonary embolism	351	4.99 (4.49, 5.55)	4.96 (4.5, 5.47)	1,106.03	2.3 (2.15)	4.94 (4.52)
Psychiatric disorders	Mental status changes	111	5.5 (4.56, 6.63)	5.49 (4.51, 6.68)	405.57	2.45 (2.18)	5.47 (4.68)
Hepatobiliary disorders	Hepatic function abnormal	106	4.2 (3.47, 5.08)	4.19 (3.44, 5.1)	256.66	2.06 (1.79)	4.18 (3.56)
Congenital, familial and genetic disorders	Hypermutation	88	52,495.77 (12,924.3, 213,226.75)	52,382.9 (12,773.6, 214,815.65)	102,433.9	10.19 (9.76)	1,165.04 (360.58)

### 3.4 Time to onset of TMZ-associated AEs

**FIGURE 3:**
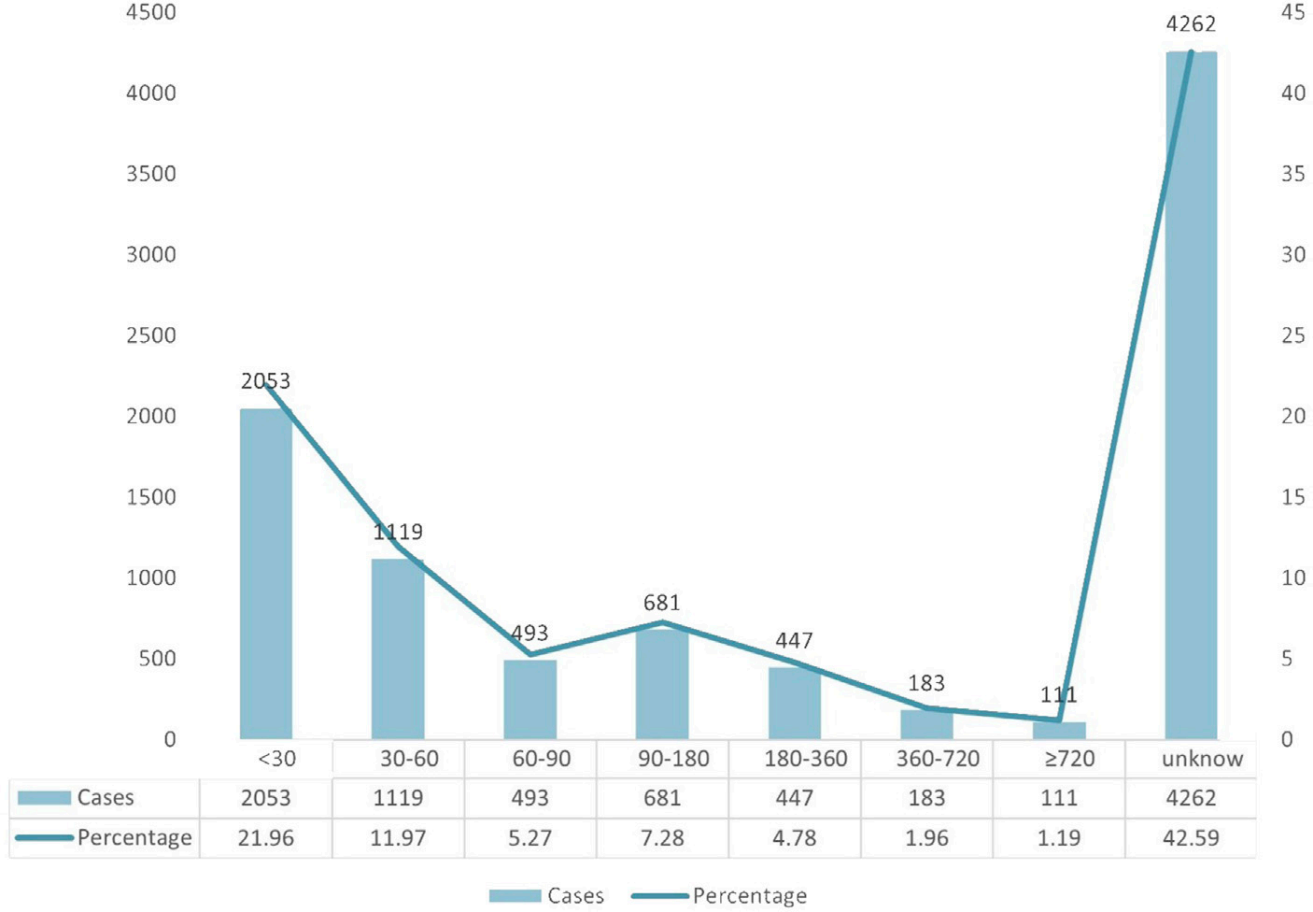
Time to onset of TMZ-associated AEs.

A total of 9,349 AE case reports were included, of which 4,262 had inaccurate or missing time of onset reports, and 5,087 had complete time of onset reports. The median and quartiles were 45.00 (20.00, 140.00). The grouping and trend of the time of onset are shown in Figure 3. Most of the adverse reactions occurred within 30 days (2053 patients, 21.96%), up to 2 months (1,119 patients, 11.97%), and up to 3 months (493 patients, 5.27%) of treatment, whereas more than half a year (447 patients, 4.78%) and more than 2 years (111 patients, 1.19%).

### 3.5 Relevant indications for the utilization of temozolomide in FAERS database

**FIGURE 4:**
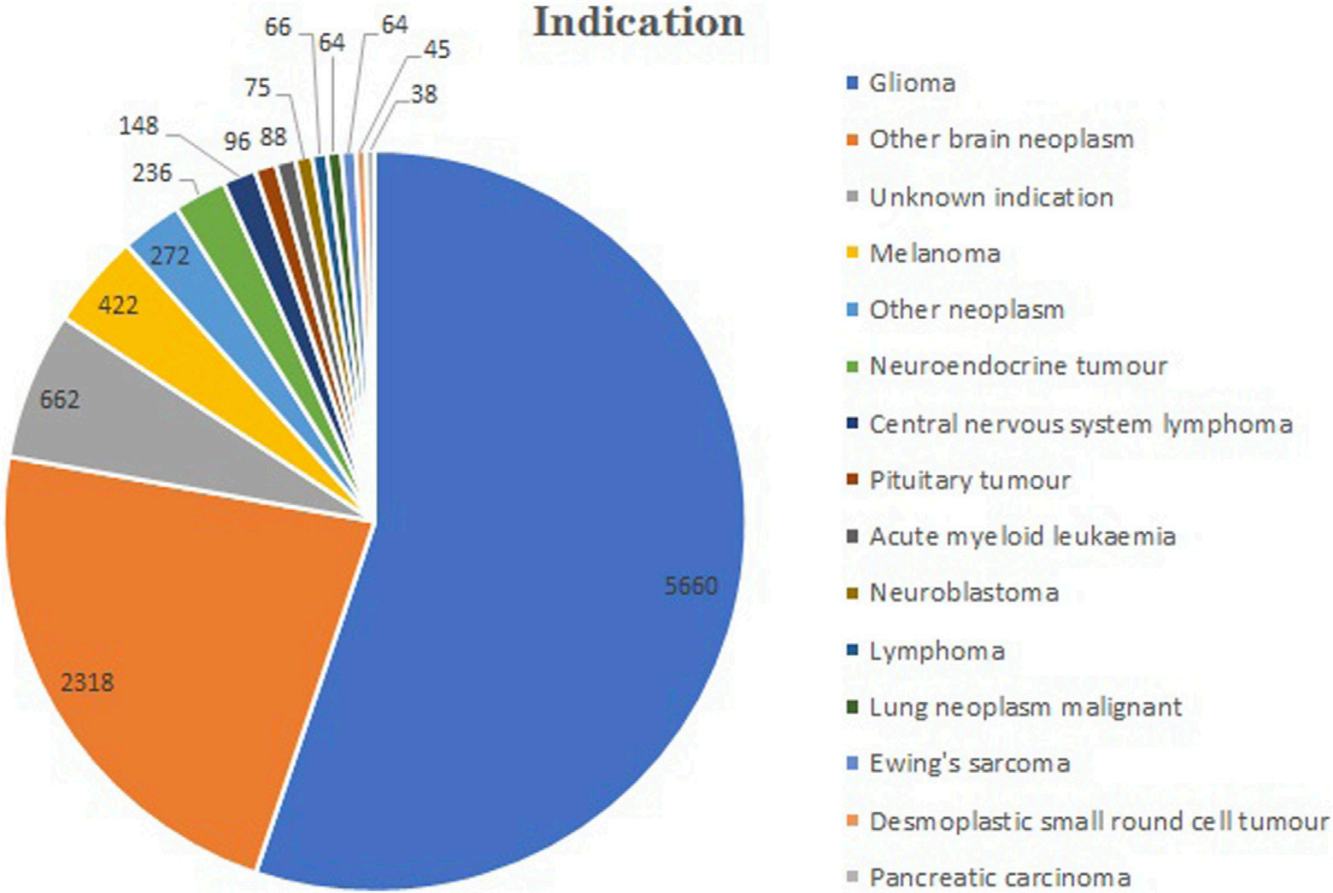
Relevant indications for the utilization of temozolomide in FAERS database.

The total number of indications for TMZ extracted from the FAERS from 2004 to 2023 was 416. A part of indications exhibited similarities despite their differing nomenclature. The top 40 indications were categorized and tallied (Figure 4). For example, we categorized all the different types of gliomas as gliomas. The five most numerous reported indications were glioma (n = 5,660), other brain neoplasm (n = 2,318), melanoma (n = 422), other neoplasm (n = 272) and neuroendocrine tumor (n = 236).

## 4 Discussion

TMZ is an antitumor drug approved by the FDA and is the most widely used agent for the treatment of glioma. As a drug precursor drug, it is gradually metabolized and completely absorbed in an acidic environment; however, it can be rapidly decomposed to form MTIC at pH > 7. It reacts with water to form AIC and methyl diazo cations. Subsequently, the methyl diazo cation is methylated at the N7 and O6 positions of guanine and the O3 position of adenine (Denny et al., 1994). During DNA replication, the methylation of O6 allows for the mismatch of guanine and thymine; additionally, after the mismatch is recognized by mismatch repair (MMR) proteins of DNA, futile attempts to repair these adducts lead to DNA double-strand breaks, which consequently trigger apoptosis (Newlands et al., 1997; Roos et al., 2007). Among these methylated DNA adducts, methylation of the O6 position of guanine is the most important mechanism of TMZ antitumor activity (Friedman et al., 2000). MGMT is a base excision repair protein that removes methyl from the O6 position of methylated guanine and inactivates it. Cells lacking the MGMT protein are more susceptible to TMZ due to methylation of the promoter region of the MGMT gene. Therefore, patients with a methylated MGMT gene promoter region in glioma cells respond better to TMZ. A previous study showed that MGMT methylation is associated with longer survival in patients treated with TMZ (Hegi et al., 2005). However, the MGMT methylation status may be associated with potential adverse effects of TMZ. Methylation of MGMT has been reported to be associated with an increased risk of hematologic adverse events (HAEs) because bone marrow precursor cells have low levels of MGMT, and the use of TMZ leads to a decrease in MGMT activity, which further contributes to HAEs (Gerson et al., 1996; Sabharwal et al., 2011). This indicate low MGMT expression in peripheral blood mononuclear cells has been associated with increased hematotoxicity following TMZ treatment. Alternatively, certain polymorphisms in MGMT may also be associated with an increased risk of HAEs (Gerson et al., 1996; Briegert et al., 2007; Drabløs et al., 2004). In a clinical trial involving 347 patients with malignant glioma treated with TMZ, the safety of TMZ was clearly evaluated, and the most common adverse effects included thrombocytopenia, anemia, nausea, vomiting, and anorexia (Bae et al., 2014). By analyzing the FAERS database from the first quarter of 2004 to the fourth quarter of 2023, this study systematically evaluated the adverse reactions associated with TMZ. Through this process, this study not only confirmed some existing safety information but also identified new potential risks. The findings presented in this study offer valuable and precise insights into the safety profile of TMZ in real-world clinical settings. The following is an in-depth discussion of the study results.

This study demonstrated that reports of AEs involving TMZ were more prevalent in male patients than in female patients. This may be related to the incidence of gliomas in males and females. According to the Surveillance Epidemiology and End Results report, the incidence of glioblastoma is 1.5 times greater in male patients than in female patients, with a male to female ratio of 3.99/100,000:2.53/100,000 (1.57) (http://www.cbtrus.org/2011-NPCR-SEER/WEB-0407-Report-3-3-2011.pdf). We also observed a greater proportion of adverse reactions to TMZ in patients aged >60 years (34.55%). This may be partly attributable to the greater risk of disease in this age group and partly to the greater resistance to ADRs in relatively younger individuals. It is worth mentioning that the majority of the AE reports were from medical professionals, including physicians (30.45%), pharmacists (27.64%), and other health professionals (13.96%), which reflects the importance that medical professionals attribute to ADR reports of TMZ. The number of AEs in the United States was significantly greater than that in other countries. This underlying trend may be attributed to factors such as stronger reporting willingness, earlier market entry, and earlier expansion of indications, which have collectively facilitated its broader usage. In terms of TMZ indications, current clinical practice primarily focuses on treating nervous system tumors such as gliomas. However, it has been noted that TMZ is also utilized to a limited extent in certain other malignant tumors, although this usage is not widely reported.

In our study, the most common and significant SOC-related AEs, such as blood and lymphatic system disorders; benign neoplasms; malignant and unspecified neoplasms; hepatobiliary disorders; congenital, familial and genetic disorders; infections and infestations; metabolic and nutritional disorders; endocrine disorders; nervous system disorders; and gastrointestinal disorders, were consistent with the safety data from labeling and clinical trials. Blood and lymphatic system disorders are by far the most reported TMZ-related ADRs. Among the PTs corresponding to blood and lymphatic system disorders, the four most commonly reported PTs were those with thrombocytopenia, neutropenia, pancytopenia and anemia. This finding is consistent with the conclusions reported in multiple clinical trials (Bae et al., 2014; Garcia et al., 2022; Kesari et al., 2009). The most common AEs of TMZ also included gastrointestinal disorders and hepatobiliary disorders. In terms of gastrointestinal disorders and hepatobiliary disorders at the PT level, nausea, vomiting, constipation, and elevations in the Alt, Ast, and GGP were the most commonly reported. Our results were further validated in clinical trials and drug packages (Bae et al., 2014). Such common AEs tend to be tolerable and of low grade. In a study by Bae et al. (2014), 84.8% of the 618 toxicities were Common Terminology Criteria for Adverse Events (CTCAE) grade 1 or 2, while a total of 15.2% of the patients were grade 3 or 4, and there were no deaths. Among the HAEs, thrombocytopenia (13.7%), anemia (11.0%), and increased AST/ALT (7.0%) were commonly observed. Among the nonhematologic toxicities, nausea (44.3%), vomiting (37.0%), and anorexia (14.3%) were the three most common side effects. In the study by Garcia et al., which included 1,454 patients treated with temozolomide, the median survival times were 18.6 months for patients who presented with anemia, 20.7 months for those with leukopenia, 18.5 months for those with lymphopenia, 19.5 months for those with neutropenia, and 16.5 months for those with thrombocytopenia (Garcia et al., 2022). It can be concluded that such common TMZ-related HAEs have no significant effect on the prognosis of patients. Myelosuppression, aplastic anemia, and myelodysplastic syndromes were also reported in our study. Among alkylating agents, aplastic anemia appears to be unique to TMZ, and the first report of aplastic anemia associated with TMZ was published in 2006 (Villano et al., 2006). Multiple reports of aplasticanemia, myelodysplastic syndrome, and leukemia, as well as severe myelosuppression, have subsequently appeared (Chamberlain and Raizer, 2009; De Vita et al., 2005; George et al., 2009; Jalali et al., 2007; Singhal et al., 2007; Noronha et al., 2006). Dixit et al. referred to these AEs as being hematological idiosyncratic drug reactions (IDRs), and TMZ-related hematological IDRs, although rare, are associated with significant mortality. This study analyzed 21 IDRs, including aplastic anemia (n = 11), severe myelosuppression (n = 4), and bone marrow insufficiency (n = 6). All of the patients received TMZ in combination with radiotherapy, and after 4 weeks, 60% (13/21) of the patients died of septicemia or internal hemorrhage (Dixit et al., 2012). The occurrence of IDRs is often associated with cytogenetic abnormalities. Acquired aplastic anemia may be linked to frequent loss of HLA alleles associated with copy number-neutral 6p arms loss of heterozygosity (Katagiri et al., 2011). TMZ-related myelodysplastic syndrome and acute myeloid leukemia frequently exhibit chromosomal deletions and translocations (Su et al., 2005; Kim et al., 2009; Pedersen-Bjergaard, 2005). Armstrong et al. identified clinical factors for TMZ-related HAEs and proposed a model to predict the occurrence of HAEs (Armstrong et al., 2009). The incidence of HAEs is higher in women than in men. This is consistent with our analysis (Supplementary Table S4). For males, BSA > 2 m^2^, not being on steroids, and taking bowel medication were associated with an increased risk of HAE. For females, the following were associated with an increased risk of HAE: no prior chemotherapy, baseline creatinine >1 mg/dL, baseline platelet count <270,000/mm^3^, BSA <2 m^2^, being on analgesics, and not being on medication for gastroesophageal reflux disease (Armstrong et al., 2009). Common TMZ-related HAEs are usually mild and reversed after discontinuation or decreased TMZ dose without the need for further intervention, and even the occurrence of HAEs during chemotherapy is associated with a better prognosis (Garcia et al., 2022). The research conducted by Ingham et al. (2023) also confirmed that myelosuppression was manageable with dose alteration and did not result in clinically significant AEs. However, hematological IDRs have a fatal effect on patients. Therefore, a cyclical assessment of hematopoietic function should be implemented during treatment of TMZ. Monitoring hematological indicators should not be limited, and timely intervention to avoid the occurrence of hematological IDRs is essential for optimizing the application of TMZ, thus improving the safety of TMZ and reducing patient mortality.

Among the PTs corresponding to nervous system disorders, we identified signs such as seizures, cerebral edema, unilateral paresis, aphasia, cerebral hemorrhage and hydrocephalus. Except for unilateral paresis, the other AEs have never been described on the drug label and have rarely been reported in standardized trials. ACT IV was a large, randomized, double-blind, international phase III trial that included 745 patients with glioblastoma receiving adjuvant therapy with TMZ, with neurological-related AEs reported in the study, including cerebral edema (38/745, 5.1%), epilepsy (57/745, 7.7%) and headache (16/745, 2.1%) (Weller et al., 2017). The mechanism underlying TMZ-related seizures remains unclear. However, it is worth conducting further investigations to elucidate the potential association, considering the common occurrence of tumor-related seizures in glioblastoma patients. Notably, current study demonstrates a significant reduction in seizure incidence with increased utilization of TMZ (Koekkoek et al., 2015). In terms of metabolism and nutritional disorders at the PT level, we observed off-label ADRs such as dehydration, hyponatremia, hyperglycemia, and hypokalemia. The results of a phase 2 clinical trial in children showed that 6 of the 35 children enrolled in the study who received TMZ-assisted therapy developed hypokalemia (Mody et al., 2017). Two other studies of TMZ in children also reported of AEs of hyperkalemia (Robison et al., 2018; Wagner et al., 2010). This may suggest that TMZ-associated hypokalemia often occurs in younger individuals. Hyponatremia is often accompanied by hypotonic dehydration, and two studies on the diagnosis and management of melanoma reported of TMZ-associated hyponatremia, which may indicate that hyponatremia is relevant to the use of TMZ in melanoma (Ott et al., 2013; Bilir et al., 2016). The most common AEs that we observed in infections and infestations included sepsis, Aspergillus infection, infectious shock, and infectious aspiration pneumonia. Infectious shock is a serious complication of sepsis, and the development of sepsis is often associated with severe HAEs caused by TMZ. When infection occurs in patients with aplastic anemia, severe granulocytopenia, or severe myelosuppression, the body’s immune function is low, and its ability to clear pathogens is weakened, thus leading to massive multiplication of pathogens in the body, which consequently makes the body more prone to progression to sepsis. Two studies reported of two cases of sepsis triggered after TMZ-associated neutropenia with concomitant infection (Gutiérrez Pérez et al., 2023; Özdirik et al., 2021). Aspergillus infection is associated with a risk of TMZ-induced opportunistic infections. This may be attributed to T lymphocyte immune dysfunction resulting from TMZ-induced immunosuppression. Moreover, the concurrent administration of corticosteroids for glioma treatment to alleviate brain edema further augments the susceptibility to infection. Despite the absence of substantial evidence from large-scale clinical trials, a number of case reports have indicated a potential correlation between TMZ and opportunistic infections (Brault et al., 2021; Damek et al., 2008). Clinicians should be cognizant of the potent immunosuppressive effects of TMZ in combination with corticosteroids. Vigilance towards infection symptoms, timely utilization of NGS or microbiome biomarkers for early detection, and prompt treatment of the underlying cause can prevent the occurrence of severe infectious complications (Liu et al., 2019).

One of the most common AE signals that we have detected in congenital, familial and genetic disorders is hypermutation. Hypermutation in TMZ may account for the growing number of patients with treatment-related AML (tAML) and MDS. tAML is reported in 3%–10% of patients who receive alkylating agents for Hodgkin lymphoma, non-Hodgkin lymphoma, ovarian cancer, breast cancer, or multiple myeloma (Seedhouse and Russell, 2007). The MMR gene is closely related to TMZ-associated hypermutation, and a previous study confirmed that MMR gene deficiency leads to hypermutation and chemoresistance in gliomas (Touat et al., 2020). This scenario may be related to base mismatches induced by TMZ hydrolysis products and DNA double-strand breaks caused by the failure of homologous repair after mismatches, which further altered the genes of the tumor. Moreover, TMZ-associated hypermutation may be associated with malignant transformation of low-grade gliomas. Johnson et al. compared the mutational profiles of 23 IDH-mutant, low-grade astrocytomas at initial diagnosis versus at tumor recurrence to determine the extent to which mutations in the initial tumors differ from their subsequent recurrent tumors and how treatment with TMZ affects the mutational profile (Johnson et al., 2014). Among the 10 TMZ-treated low-grade astrocytomas, 6 exhibited TMZ-induced hypermutation, and all 6 hypermutated tumors underwent malignant transformation to glioblastoma (Johnson et al., 2014). Another study also showed that TMZ-induced hypermutation is associated with distant recurrence and reduced survival after high-grade transformation of low-grade IDH-mutant gliomas (Yu et al., 2021). Therefore, an understanding of the risk of TMZ-induced hypermutation is critical for patients and clinicians to accurately assess the risks and benefits of TMZ therapy. The mechanisms and causes of TMZ-induced malignancies are currently unclear. Although low-grade gliomas are susceptible to TMZ, it is not clear whether this is limited to certain low-grade gliomas, and further studies are needed to clarify the therapeutic impact of TMZ-associated hypermutation. Other unreported AEs, such as petechiae, deep vein thrombosis and pulmonary embolism, may be caused by direct toxicity or allergic reactions to the drug. However, the exact underlying mechanism remains to be further explored. These findings suggest the need for closer monitoring of electrolytes, infection markers, and hematologic markers in patients treated with TMZ, as well as vigilance regarding the risk of carcinogenesis due to hypermutation. Symptom interventions should be performed when needed. These findings emphasize the importance of continuous monitoring of drug-related AEs and provide a valuable reference for informed decision-making in drug selection.

Although AEs, including psychiatric disorders, fatigue, visual abnormalities, hearing impairments, cough, palpitations, alopecia, and nephrotoxicity, have been documented in certain clinical trials and drug descriptions, our comprehensive data analyses did not demonstrate any noteworthy signals of these specific ADRs. We believe that this discrepancy may be attributable to reporting biases between different reporting groups. In clinical trials, AEs are recorded according to uniform and strict criteria, whereas reporting in the FAERS database is voluntary, which may have led to a large number of unnoticed or low-impact AEs being overlooked, thus contributing to the bias in our study. However, the advantages of the large-scale and real-world nature of the FAERS database are significant, thus allowing for a more complete narrative and observation to recognize the AEs and clinical use of TMZ and providing us with more AEs that have not yet been reported. Thus, these findings could add considerable evidence of the practicality of TMZ to the safety data and provide new perspectives for improving the clinical use of this drug. Our study encompasses the broadest set of cases associated with TMZ to date; in addition to consolidating previously cataloged ADRs consistent with drug labels and established clinical trials, we also identified a number of new and unexpected significant AEs. In addition, we analyzed the adverse effects that we observed, including severity, proportion, and impact on clinical prognosis. All of these findings provide comprehensive and valuable insights into the safety of TMZ.

This research had several limitations. First, the FAERS database operates as a self-reporting system and is susceptible to underreporting, duplicate reporting, and inaccurate reporting, all of which have the potential to introduce bias into the study findings. Second, the FAERS database includes only data on AEs reported over a period of time since the drug’s introduction to the market; thus, without an assessment of the severity of these AEs, only qualitative evaluations of these AEs could be made. Third, the specificity of TMZ-induced AEs is limited and may be influenced by concurrent administration. Furthermore, the absence of age-specific details in a significant portion of the data hinders our comprehensive understanding of the occurrence of AEs across different age groups. Future research should prioritize the acquisition of precise age-related information and investigate potential variations in ADRs associated with different age brackets. In conclusion, this study represents a significant exploration within the field of signal mining. Despite its limitations, the findings underscore the necessity for rigorous follow-up monitoring and further investigation through case‒control studies. Additionally, they offer a pharmacovigilance perspective that can contribute to the treatment of gliomas and the utilization of TMZ.

## 5 Conclusion

Our study represents the first comprehensive and detailed pharmacovigilance analysis of TMZ utilization utilizing real-world data from the FAERS database. Our findings elucidate the characteristics of hematologic IDRs, such as aplastic anemia, severe myelosuppression and myelodysplastic syndrome, and provide initial insights into the relationship between HAEs and infectious diseases. This study contributes to a deeper understanding of the interplay between AEs in different physiological systems induced by the same drug. Furthermore, our investigation demonstrated potential signals for nervous system-related AEs, including cerebral edema, epilepsy, aphasia, cerebral hemorrhage, and hydrocephalus. Additionally, it underscores the importance for medical practitioners to monitor patients’ hematologic indices, electrolyte levels and nutritional status during TMZ treatment to prevent electrolyte imbalances and hematologic IDRs. The mutagenicity of TMZ is a crucial consideration, as evidenced by numerous studies demonstrating its significant impact on tumor progression and the development of drug resistance. This warrants careful clinical attention and further in-depth basic research to elucidate the underlying mechanisms and relationships, thus ultimately leading to enhanced efficacy of TMZ and a reduced incidence of AEs. Although there are limitations in the reporting of AEs through the FAERS system, it is undeniable that the algorithm-based detection of AEs signals offers a novel perspective for clinical cognition. These new perspectives contribute to a more refined comprehension and facilitate exploration without inducing confusion. In order to establish a clearer understanding of the relationship between these adverse events and drugs, it is imperative to conduct multi-center prospective clinical trials in the future. The future research will primarily focus on investigating the mechanisms of TMZ-related hematotoxicity and neurotoxicity at the cellular and genetic levels, aiming to Improve usage in order to mitigate the risk of these severe adverse effects. In conclusion, the use of TMZ is associated with numerous unforeseen AEs and potential genotoxicity. Through pharmacovigilance research, the AEs of TMZ were comprehensively examined for the first time at both the systemic and individual levels, thus providing new insights and ideas for enhancing the development and clinical application of future drugs.

## Data Availability

The original contributions presented in the study are included in the article/Supplementary Material, further inquiries can be directed to the corresponding author.

## Abbreviations

AEs, Adverse events; FAERS, FDA Adverse Event Reporting System; PTs, preferred terms; SOCs, system organ classes; ADR, adverse drug reaction; TMZ, Temozolomide; BCPNN, Bayesian confidence propagation neural network; EBGM, empirical Bayesian geometric mean; MTIC, 5-(3-methyltriazen-1-yl) imidazole-4-carboxamide; AIC, 5-aminoimidazole-4-carboxamide; DPA, disproportionality analysis; RORs, reporting of dominance ratios; PRRs, proportional reporting ratios; HDI, Human Development Index; MMR, mismatch repair; MGMT, O6-methylguanine-DNA methyltransferase; HAEs, hematologic adverse events; IDRs, idiosyncratic drug reactions.

## References

[B1] Armstrong T. S. Cao Y. Scheurer M. E. Vera-BolañOS E. Manning R. Okcu M. F. (2009). Risk analysis of severe myelotoxicity with temozolomide: the effects of clinical and genetic factors. Neuro-oncology 11, 825–832. 10.1215/15228517-2008-120 19179423 PMC2802402

[B2] Bae S. H. Park M.-J. Lee M. M. Kim T. M. Lee S.-H. Cho S. Y. (2014). Toxicity profile of temozolomide in the treatment of 300 malignant glioma patients in Korea. J. Korean Med. Sci. 29, 980–984. 10.3346/jkms.2014.29.7.980 25045231 PMC4101787

[B3] Bilir S. P. Ma Q. Zhao Z. Wehler E. Munakata J. Barber B. (2016). Economic burden of toxicities associated with treating metastatic melanoma in the United States. Am. Health and Drug Benefits 9, 203–213. 27688833 PMC5004818

[B4] Brault C. Zerbib Y. Chouaki T. Maizel J. Nyga R. (2021). Temozolomide is a risk factor for invasive pulmonary aspergillosis: a case report and literature review. Infect. Dis. Now. 51, 630–632. 10.1016/j.idnow.2020.11.009 34581280

[B5] Briegert M. Enk A. H. Kaina B. (2007). Change in expression of MGMT during maturation of human monocytes into dendritic cells. DNA Repair 6, 1255–1263. 10.1016/j.dnarep.2007.02.008 17382605

[B6] Chamberlain M. C. Raizer J. (2009). Extended exposure to alkylator chemotherapy: delayed appearance of myelodysplasia. J. Neuro-oncology 93, 229–232. 10.1007/s11060-008-9764-5 19099199

[B7] Damek D. M. Lillehei K. O. Kleinschmidt-Demasters B. K. (2008). Aspergillus terreus brain abscess mimicking tumor progression in a patient with treated glioblastoma multiforme. Clin. Neuropathol. 27, 400–407. 10.5414/npp27400 19130738

[B8] Denny B. J. Wheelhouse R. T. Stevens M. F. Tsang L. L. Slack J. A. (1994). NMR and molecular modeling investigation of the mechanism of activation of the antitumor drug temozolomide and its interaction with DNA. Biochemistry 33, 9045–9051. 10.1021/bi00197a003 8049205

[B9] De Vita S. DE Matteis S. Laurenti L. Chiusolo P. Reddiconto G. Fiorini A. (2005). Secondary Ph+ acute lymphoblastic leukemia after temozolomide. Ann. Hematol. 84, 760–762. 10.1007/s00277-005-1093-6 16044311

[B10] Dixit S. Baker L. Walmsley V. Hingorani M. (2012). Temozolomide-related idiosyncratic and other uncommon toxicities: a systematic review. Anti-Cancer Drugs 23, 1099–1106. 10.1097/CAD.0b013e328356f5b0 22850321

[B11] Drabløs F. Feyzi E. Aas P. A. Vaagbø C. B. Kavli B. Bratlie M. S. (2004). Alkylation damage in DNA and RNA--repair mechanisms and medical significance. DNA Repair 3, 1389–1407. 10.1016/j.dnarep.2004.05.004 15380096

[B12] Du Y. Zhu J. Guo Z. Wang Z. Wang Y. Hu M. (2024). Metformin adverse event profile: a pharmacovigilance study based on the FDA Adverse Event Reporting System (FAERS) from 2004 to 2022. Expert Rev. Clin. Pharmacol. 17, 189–201. 10.1080/17512433.2024.2306223 38269492

[B13] Fang C. Wang K. Stephen Z. R. Mu Q. Kievit F. M. Chiu D. T. (2015). Temozolomide nanoparticles for targeted glioblastoma therapy. ACS Appl. Mater. and Interfaces 7, 6674–6682. 10.1021/am5092165 25751368 PMC4637162

[B14] Friedman H. S. Kerby T. Calvert H. (2000). Temozolomide and treatment of malignant glioma. Clin. Cancer Res. 6, 2585–2597. 10914698

[B15] Garcia C. R. Myint Z. W. Jayswal R. Wang C. Morgan R. M. Butts A. R. (2022). Hematological adverse events in the management of glioblastoma. J. Neuro-Oncology 156, 153–161. 10.1007/s11060-021-03891-8 PMC8829911 34820776

[B16] George B. J. Eichinger J. B. Richard T. J. (2009). A rare case of aplastic anemia caused by temozolomide. South. Med. J. 102, 974–976. 10.1097/SMJ.0b013e3181b1d2fa 19668033

[B17] Gerson S. L. Phillips W. Kastan M. Dumenco L. L. Donovan C. (1996). Human CD34+ hematopoietic progenitors have low, cytokine-unresponsive O6-alkylguanine-DNA alkyltransferase and are sensitive to O6-benzylguanine plus BCNU. Blood 88, 1649–1655. 10.1182/blood.v88.5.1649.1649 8781420

[B18] Gutiérrez Pérez C. Chivato MartíN-Falquina I. RodríGUEZ Ledesma I. Cuenca Zarzuela A. Soto Alsar J. Juliao CaamañO D. S. (2023). Sepsis due to phlegmonous gastritis in a cancer patient. Rev. Espanola Enfermedades Dig. 115, 143–144. 10.17235/reed.2022.9009/2022 35791795

[B19] Hegi M. E. Diserens A.-C. Gorlia T. Hamou M.-F. DE Tribolet N. Weller M. (2005). MGMT gene silencing and benefit from temozolomide in glioblastoma. N. Engl. J. Med. 352, 997–1003. 10.1056/NEJMoa043331 15758010

[B20] Ingham M. Allred J. B. Chen L. Das B. Kochupurakkal B. Gano K. (2023). Phase II study of olaparib and temozolomide for advanced uterine leiomyosarcoma (NCI protocol 10250). J. Clin. Oncol. 41, 4154–4163. 10.1200/JCO.23.00402 37467452 PMC10852403

[B21] Iturrioz-RodríGUEZ N. Sampron N. Matheu A. (2023). Current advances in temozolomide encapsulation for the enhancement of glioblastoma treatment. Theranostics 13, 2734–2756. 10.7150/thno.82005 37284445 PMC10240814

[B22] Jalali R. Singh P. Menon H. Gujral S. (2007). Unexpected case of aplastic anemia in a patient with glioblastoma multiforme treated with Temozolomide. J. Neuro-Oncology 85, 105–107. 10.1007/s11060-007-9398-z 17505778

[B23] Johnson B. E. Mazor T. Hong C. Barnes M. Aihara K. Mclean C. Y. (2014). Mutational analysis reveals the origin and therapy-driven evolution of recurrent glioma. Sci. (New York, N.Y.) 343, 189–193. 10.1126/science.1239947 PMC3998672 24336570

[B24] Katagiri T. Sato-Otsubo A. Kashiwase K. Morishima S. Sato Y. Mori Y. (2011). Frequent loss of HLA alleles associated with copy number-neutral 6pLOH in acquired aplastic anemia. Blood 118, 6601–6609. 10.1182/blood-2011-07-365189 21963603

[B25] Kesari S. Schiff D. Drappatz J. Lafrankie D. Doherty L. Macklin E. A. (2009). Phase II study of protracted daily temozolomide for low-grade gliomas in adults. Clin. Cancer Res. Official J. Am. Assoc. For Cancer Res. 15, 330–337. 10.1158/1078-0432.CCR-08-0888 19118062

[B26] Kim S. J. Park T. S. Lee S. T. Song J. Suh B. Kim S. H. (2009). Therapy-related myelodysplastic syndrome/acute myeloid leukemia after treatment with temozolomide in a patient with glioblastoma multiforme. Ann. Clin. Laboratory Sci. 39, 392–398. 19880768

[B27] Koekkoek J. A. F. Dirven L. Heimans J. J. Postma T. J. Vos M. J. Reijneveld J. C. (2015). Seizure reduction in a low-grade glioma: more than a beneficial side effect of temozolomide. J. Neurology, Neurosurg. Psychiatry 86, 366–373. 10.1136/jnnp-2014-308136 25055819

[B28] Lee S. Y. (2016). Temozolomide resistance in glioblastoma multiforme. Genes and Dis. 3, 198–210. 10.1016/j.gendis.2016.04.007 PMC6150109 30258889

[B29] Liu S. A. Sullivan T. Bryce C. Chan A. M. Cilmi S. (2019). Cerebral aspergillosis within new tumour site presents as incidental new brain lesion in patient receiving temozolomide for glioblastoma multiforme. BMJ Case Rep. 12, e227500. 10.1136/bcr-2018-227500 PMC6557343 31154345

[B30] Mccormack A. (2022). Temozolomide in aggressive pituitary tumours and pituitary carcinomas. Best Pract. and Res. Clin. Endocrinol. and Metabolism 36, 101713. 10.1016/j.beem.2022.101713 36274026

[B31] Middleton M. R. Grob J. J. Aaronson N. Fierlbeck G. Tilgen W. Seiter S. (2000). Randomized phase III study of temozolomide versus dacarbazine in the treatment of patients with advanced metastatic malignant melanoma. J. Clin. Oncol. Official J. Am. Soc. Clin. Oncol. 18, 158–166. 10.1200/JCO.2000.18.1.158 10623706

[B32] Mody R. Naranjo A. Van Ryn C. Yu A. L. London W. B. Shulkin B. L. (2017). Irinotecan-temozolomide with temsirolimus or dinutuximab in children with refractory or relapsed neuroblastoma (COG ANBL1221): an open-label, randomised, phase 2 trial. Lancet. Oncol. 18, 946–957. 10.1016/S1470-2045(17)30355-8 28549783 PMC5527694

[B33] Newlands E. S. Stevens M. F. Wedge S. R. Wheelhouse R. T. Brock C. (1997). Temozolomide: a review of its discovery, chemical properties, pre-clinical development and clinical trials. Cancer Treat. Rev. 23, 35–61. 10.1016/s0305-7372(97)90019-0 9189180

[B34] Noronha V. Berliner N. Ballen K. K. Lacy J. Kracher J. Baehring J. (2006). Treatment-related myelodysplasia/AML in a patient with a history of breast cancer and an oligodendroglioma treated with temozolomide: case study and review of the literature. Neuro-oncology 8, 280–283. 10.1215/15228517-2006-003 16728498 PMC1871950

[B35] Ott P. A. Chang J. Madden K. Kannan R. Muren C. Escano C. (2013). Oblimersen in combination with temozolomide and albumin-bound paclitaxel in patients with advanced melanoma: a phase I trial. Cancer Chemother. Pharmacol. 71, 183–191. 10.1007/s00280-012-1995-7 23064957

[B36] Özdirik B. Amthauer H. Schatka I. Goretzki P. E. Mogl M. T. Fehrenbach U. (2021). A rare case of a patient with a high grade neuroendocrine tumor developing neutropenic sepsis after receiving PRRT combined with Capecitabine or Temozolomide: a case report. Mol. Clin. Oncol. 14, 20. 10.3892/mco.2020.2182 33363730 PMC7725216

[B37] Pedersen-Bjergaard J. (2005). Insights into leukemogenesis from therapy-related leukemia. N. Engl. J. Med. 352, 1591–1594. 10.1056/NEJMe048336 15829541

[B38] Robison N. J. Yeo K. K. Berliner A. P. Malvar J. Sheard M. A. Margol A. S. (2018). Phase I trial of dasatinib, lenalidomide, and temozolomide in children with relapsed or refractory central nervous system tumors. J. Neuro-oncology 138, 199–207. 10.1007/s11060-018-2791-y PMC5930136 29427149

[B39] Roos W. P. Batista L. F. Z. Naumann S. C. Wick W. Weller M. Menck C. F. M. (2007). Apoptosis in malignant glioma cells triggered by the temozolomide-induced DNA lesion O6-methylguanine. Oncogene 26, 186–197. 10.1038/sj.onc.1209785 16819506

[B40] Sabharwal A. Waters R. Danson S. Clamp A. Lorigan P. Thatcher N. (2011). Predicting the myelotoxicity of chemotherapy: the use of pretreatment O6-methylguanine-DNA methyltransferase determination in peripheral blood mononuclear cells. Melanoma Res. 21, 502–508. 10.1097/CMR.0b013e32832ccd58 19561552

[B41] Seedhouse C. Russell N. (2007). Advances in the understanding of susceptibility to treatment-related acute myeloid leukaemia. Br. J. Haematol. 137, 513–529. 10.1111/j.1365-2141.2007.06613.x 17539774

[B42] Singhal N. Selva-Nayagam S. Brown M. P. (2007). Prolonged and severe myelosuppression in two patients after low-dose temozolomide treatment-case study and review of literature. J. Neuro-oncology 85, 229–230. 10.1007/s11060-007-9403-6 17530175

[B43] Stupp R. Hegi M. E. Mason W. P. Van Den Bent M. J. Taphoorn M. J. B. Janzer R. C. (2009). Effects of radiotherapy with concomitant and adjuvant temozolomide versus radiotherapy alone on survival in glioblastoma in a randomised phase III study: 5-year analysis of the EORTC-NCIC trial. Lancet. Oncol. 10, 459–466. 10.1016/S1470-2045(09)70025-7 19269895

[B44] Su Y.-W. Chang M.-C. Chiang M.-F. Hsieh R.-K. (2005). Treatment-related myelodysplastic syndrome after temozolomide for recurrent high-grade glioma. J. Neuro-oncology 71, 315–318. 10.1007/s11060-004-2028-0 15735923

[B45] Sung H. Ferlay J. Siegel R. L. Laversanne M. Soerjomataram I. Jemal A. (2021). Global cancer statistics 2020: GLOBOCAN estimates of incidence and mortality worldwide for 36 cancers in 185 countries. CA a Cancer J. For Clin. 71, 209–249. 10.3322/caac.21660 33538338

[B46] Tatar Z. Thivat E. Planchat E. Gimbergues P. Gadea E. Abrial C. (2013). Temozolomide and unusual indications: review of literature. Cancer Treat. Rev. 39, 125–135. 10.1016/j.ctrv.2012.06.002 22818211

[B47] Tian X.-H. Lin X.-N. Wei F. Feng W. Huang Z.-C. Wang P. (2011). Enhanced brain targeting of temozolomide in polysorbate-80 coated polybutylcyanoacrylate nanoparticles. Int. J. Nanomedicine 6, 445–452. 10.2147/IJN.S16570 21445277 PMC3061435

[B48] Touat M. Li Y. Y. Boynton A. N. Spurr L. F. Iorgulescu J. B. Bohrson C. L. (2020). Mechanisms and therapeutic implications of hypermutation in gliomas. Nature 580, 517–523. 10.1038/s41586-020-2209-9 32322066 PMC8235024

[B49] Van Den Bent M. J. Baumert B. Erridge S. C. Vogelbaum M. A. Nowak A. K. Sanson M. (2017). Interim results from the CATNON trial (EORTC study 26053-22054) of treatment with concurrent and adjuvant temozolomide for 1p/19q non-co-deleted anaplastic glioma: a phase 3, randomised, open-label intergroup study. Lancet (London, Engl.) 390, 1645–1653. 10.1016/S0140-6736(17)31442-3 PMC5806535 28801186

[B50] Villano J. L. Collins C. A. Manasanch E. E. Ramaprasad C. Van Besien K. (2006). Aplastic anaemia in patient with glioblastoma multiforme treated with temozolomide. Lancet. Oncol. 7, 436–438. 10.1016/S1470-2045(06)70696-9 16648049

[B51] Wagner L. M. Perentesis J. P. Reid J. M. Ames M. M. Safgren S. L. Nelson M. D. (2010). Phase I trial of two schedules of vincristine, oral irinotecan, and temozolomide (VOIT) for children with relapsed or refractory solid tumors: a children’s oncology group phase I consortium study. Pediatr. Blood and Cancer 54, 538–545. 10.1002/pbc.22407 PMC3074342 20049936

[B52] Wahl M. Phillips J. J. Molinaro A. M. Lin Y. Perry A. Haas-Kogan D. A. (2017). Chemotherapy for adult low-grade gliomas: clinical outcomes by molecular subtype in a phase II study of adjuvant temozolomide. Neuro-oncology 19, 242–251. 10.1093/neuonc/now176 27571885 PMC5464133

[B53] Weller M. Butowski N. Tran D. D. Recht L. D. Lim M. Hirte H. (2017). Rindopepimut with temozolomide for patients with newly diagnosed, EGFRvIII-expressing glioblastoma (ACT IV): a randomised, double-blind, international phase 3 trial. Lancet. Oncol. 18, 1373–1385. 10.1016/S1470-2045(17)30517-X 28844499

[B54] Yu Z.-C. Li T. Tully E. Huang P. Chen C.-N. Oberdoerffer P. (2023). Temozolomide sensitizes arid1a-mutated cancers to PARP inhibitors. Cancer Res. 83, 2750–2762. 10.1158/0008-5472.CAN-22-3646 37306706 PMC10527942

[B55] Yu Y. Villanueva-Meyer J. Grimmer M. R. Hilz S. Solomon D. A. Choi S. (2021). Temozolomide-induced hypermutation is associated with distant recurrence and reduced survival after high-grade transformation of low-grade IDH-mutant gliomas. Neuro-oncology 23, 1872–1884. 10.1093/neuonc/noab081 33823014 PMC8563321

